# A Pilot randomized trial to examine effects of a hybrid closed-loop insulin delivery system on neurodevelopmental and cognitive outcomes in adolescents with type 1 diabetes

**DOI:** 10.1038/s41467-022-32289-x

**Published:** 2022-08-30

**Authors:** Allan L. Reiss, Booil Jo, Ana Maria Arbelaez, Eva Tsalikian, Bruce Buckingham, Stuart A. Weinzimer, Larry A. Fox, Allison Cato, Neil H. White, Michael Tansey, Tandy Aye, William Tamborlane, Kimberly Englert, John Lum, Paul Mazaika, Lara Foland-Ross, Matthew Marzelli, Nelly Mauras, the Diabetes Research in Children Network (DirecNet) Consortium, Gabby Tong, Hanyang Shen, Zetan Li, Ryan Kingman, Lucy Levandoski, Julie Coffey, Rachel Bisbee, Amy Stephen, Kate Weyman, Keisha Bird, Kimberly Ponthieux, Juan Marrero

**Affiliations:** 1 Center for Interdisciplinary Brain Sciences, Department of Psychiatry and Behavioral Sciences, Stanford University, Stanford, CA USA; 2 Department of Radiology, Stanford University, Stanford, CA USA; 3 Department of Pediatrics, Stanford University, Stanford, CA USA; 4 Divisions of Endocrinology & Diabetes, at Washington University in St, Louis, St, Louis, MO USA; 5 Stead Family Department of Pediatrics, Endocrinology and Diabetes, University of Iowa, Iowa City, IA USA; 6 Department of Pediatrics, Yale University, New Haven, CT USA; 7 Division of Endocrinology, Diabetes & Metabolism, Nemours Children’s Health, Jacksonville, FL USA; 8 Division of Neurology, Nemours Children’s Health, Jacksonville, FL USA; 9 Jaeb Center for Health Research, Tampa, FL USA

**Keywords:** Type 1 diabetes, Development of the nervous system

## Abstract

Type 1 diabetes (T1D) is associated with lower scores on tests of cognitive and neuropsychological function and alterations in brain structure and function in children. This proof-of-concept pilot study (ClinicalTrials.gov Identifier NCT03428932) examined whether MRI-derived indices of brain development and function and standardized IQ scores in adolescents with T1D could be improved with better diabetes control using a hybrid closed-loop insulin delivery system. Eligibility criteria for participation in the study included age between 14 and 17 years and a diagnosis of T1D before 8 years of age. Randomization to either a hybrid closed-loop or standard diabetes care group was performed after pre-qualification, consent, enrollment, and collection of medical background information. Of 46 participants assessed for eligibility, 44 met criteria and were randomized. Two randomized participants failed to complete baseline assessments and were excluded from final analyses. Participant data were collected across five academic medical centers in the United States. Research staff scoring the cognitive assessments as well as those processing imaging data were blinded to group status though participants and their families were not. Forty-two adolescents, 21 per group, underwent cognitive assessment and multi-modal brain imaging before and after the six month study duration. HbA1c and sensor glucose downloads were obtained quarterly. Primary outcomes included metrics of gray matter (total and regional volumes, cortical surface area and thickness), white matter volume, and fractional anisotropy. Estimated power to detect the predicted treatment effect was 0.83 with two-tailed, α = 0.05. Adolescents in the hybrid closed-loop group showed significantly greater improvement in several primary outcomes indicative of neurotypical development during adolescence compared to the standard care group including cortical surface area, regional gray volumes, and fractional anisotropy. The two groups were not significantly different on total gray and white matter volumes or cortical thickness. The hybrid closed loop group also showed higher Perceptual Reasoning Index IQ scores and functional brain activity more indicative of neurotypical development relative to the standard care group (both secondary outcomes). No adverse effects associated with study participation were observed. These results suggest that alterations to the developing brain in T1D might be preventable or reversible with rigorous glucose control. Long term research in this area is needed.

## Abstract (web summary)

Children with type 1 diabetes (T1D) are at risk for reduced cognitive ability and atypical brain development. This study shows that brain and cognitive measures can be improved in adolescents with T1D using a semi-automated insulin delivery system.

## Introduction

Multiple studies have documented the potentially deleterious effects of type 1 diabetes (T1D) on the human brain. The detrimental effects of hypoglycemia are well known, especially in children whose developing brains are highly vulnerable to hypoglycemic insult^1,2^. Though near normalization of glucose and HbA1c values in children are considered the gold standard in clinical care, such goals have been very difficult to achieve in practice. As a result, clinical care of pediatric patients with T1D over the past two decades evolved to include tolerance of mild to moderate hyperglycemia, particularly during sleep when detection and treatment of early signs of hypoglycemia are often difficult^3,4^.

As clinical care of children with T1D increasingly emphasized reducing episodes of acute hypoglycemia, questions arose about the potential negative sequelae of long-term, mild-to-moderate hyperglycemia. The extant literature indicates that hyperglycemia in children with T1D is associated with lower standardized IQ and neuropsychological test scores, problems with attention, executive and psychosocial function, and long-term differences in brain structure and function^5–10^. Moreover, we recently completed a longitudinal study of 144 children with T1D and 72 age- and sex-matched typically developing children without diabetes as a component of our Diabetes Research in Children Network (DirecNet) investigation. Participants were between the ages of 4 and 9 years when recruited and were followed over four successive time points spanning 5–7 years. Lower scores on standardized measures of cognitive function were observed in the T1D group throughout the study accompanied by between-group differences in both total and regional gray and white-matter volumes, cortical thickness and area, microstructural properties of white-matter tracts, structural and functional brain connectivity, and functional brain activity^11–17^. Differences between children with T1D and the comparison group without diabetes for some brain measures were observed to widen over time^18^. In the T1D group persistent elevation of blood sugars was observed, with 50% of sensor glucose greater than the target (i.e., >180 mg/dl) at all time points suggesting continuing metabolic insult to the developing brain over time. Measures of hyperglycemia were correlated with both cognitive and brain variables^18^. Results from our longitudinal study indicate that detectable changes in brain structure and function and cognitive function arise early and persist over time in children with early-onset diabetes. These changes appear to be primarily associated with hyperglycemia.

We designed the current proof-of-concept pilot study with the principal aim to investigate whether MRI metrics of gray and white-matter development in adolescents with early-onset T1D can be improved with rigorous diabetes control using currently available, hybrid closed-loop insulin delivery diabetes technology. Forty-six adolescents ranging in age from 14 to 17 years with T1D since before age 8 years and on insulin therapy (either multiple daily injections (MDI) or open-loop pumps) were recruited. Informed written consent was obtained from the parents/guardians and the child’s assent was obtained as per local guidelines. After a run-in phase to collect baseline glucose sensor data, 44 participants who met the eligibility criteria were randomized to use either a hybrid closed-loop device with 24-hour sensor-augmented therapy (CL group; Medtronic MiniMed 670G^®^ insulin pump) or standard care (SC group; either MDI or open-loop pump). Participants not previously wearing a continuous glucose monitor (CGM) wore a blinded Medtronic iPro^®^2 Professional CGM device at baseline, 3 and 6 months for at least 6 days or continued to use their home unblinded CGM (Dexcom G5 or G6) over a 6-month period. Detailed patient instructions on device use were provided by team personnel with expertise in diabetes management and technology. Glucose and device use data were collected by downloading the CGM at 0, 3, and 6 months in both groups. All participants were administered the same standardized cognitive assessment and multimodal brain imaging evaluation utilized in our longitudinal study^18^. Full clinical, cognitive, and imaging evaluations were obtained at baseline and 6 months with repeated measurement of glycemic status using downloaded sensor data and HbA1c at baseline, 3 months, and 6 months. Of the 44 randomized participants, 42 completed the required baseline procedures and were assigned to either the SC or the CL condition. These participants were included in the final analyses following the intention-to-treat principle using mixed-effects modeling, and statistical procedures available in standard image analysis software. Our primary hypothesis was that greater reduction of hyperglycemia in the CL group, relative to the SC group, would result in greater improvement in key brain metrics—total/regional gray and white matter, cortical surface area and thickness, white-matter microstructure (fractional anisotropy)—indicative of neurotypical development during adolescence^19–23^. The secondary hypothesis was that the CL group would show higher scores on a standardized IQ assessment and functional brain activity more indicative of neurotypical development relative to the SC group. Finally, we conducted post hoc analyses to determine if improvements in key indices of hyperglycemia, specifically, time in range (glucose between 70 and 180 mg/dl) and % glucose >250 mg/dl within the entire participant cohort (i.e., regardless of group assignment) would be associated with improvement in brain and cognitive metrics. Nighttime glucose sensor measurements were emphasized in these analyses as this is the period when glucose concentrations are most likely to improve while using a hybrid closed-loop system.

## Results

### Recruitment and participant flow

Participant enrollment and follow-up took place from March 2018 through June 2020. A total of 46 participants were enrolled. Two enrolled participants did not meet inclusion/exclusion criteria and were screen failures before randomization. Two additional participants withdrew after randomization but before completing all required baseline assessments or procedures. The remaining 42 participants completed the study and were included in all data analyses in line with the intention-to-treat principle. There were no additional participant losses. The study was ended once all 42 participants completed their 6-month assessment.

### Demographic/glycemic variables

Key glycemic data at baseline and 6 months are shown in Table 1. Between-group changes in HbA1c% (and annualized HbA1c AUC% over 6 months) were not statistically significant. However, participants in the CL group showed greater overall improvement in glycemia over the 6-month period on both full-day and nighttime sensor values, as well as less glucose variation than the SC group. The percentage time in hypoglycemia (glucose <70 mg/dl) was very low for both groups.

**Table 1:** Changes in glycemic variables from baseline to 6 months and intention-to-treat (ITT) effects on these changes (all estimated based on mixed-effects modeling)

	Standard care						Closed loop						Group difference in change			
	Baseline	6 months	Change	95% CI	Effect size*	*P* value	Baseline	6 months	Change	95% CI	Effect size*	*P* value	Change	95% CI	Effect size*	*P* value
HbA1c%	8.45	8.13	−0.32	−0.70, 0.06	−0.51	0.100	8.70	8.13	−0.57	−1.06, −0.08	−0.72	0.022	−0.25	−0.87, 0.37	−0.25	0.424
% TIR	42.72	44.45	1.72	−3.83, 7.28	0.19	0.543	39.60	57.75	18.15	12.59, 23.72	2.00	0.000	16.43	8.58, 24.28	1.28	0.000
% TIR nighttime	40.09	43.00	2.91	−4.63, 10.45	0.24	0.449	39.95	64.11	24.16	16.82, 31.49	2.02	0.000	21.24	10.71, 31.78	1.23	0.000
Glucose mean (mg/dl)	196.35	195.78	−0.57	−11.72, 10.57	−0.03	0.920	205.84	173.09	−32.75	−45.82, −19.69	−1.53	0.000	−32.18	−49.27, −15.09	−1.15	0.000
% Glucose >250 mg/dl	25.81	26.37	0.56	−4.51, 5.62	0.07	0.830	29.12	14.26	−14.87	−21.33, −8.41	−1.41	0.000	−15.42	−23.60, −7.24	−1.15	0.000
% Glucose >250 mg/dl nighttime	24.73	25.49	0.75	−5.31, 6.82	0.08	0.808	23.71	12.20	−11.52	−17.83, −5.20	−1.12	0.000	−12.27	−21.02, −3.52	−0.86	0.006
% Glucose <70 mg/dl	3.70	3.35	−0.35	−1.92, 1.22	−0.14	0.664	3.34	3.88	0.54	−0.68, 1.76	0.27	0.385	0.89	−1.10, 2.88	0.27	0.381
% Glucose <70 mg/dl nighttime	7.02	5.74	−1.28	−4.06, 1.50	−0.28	0.368	5.50	2.85	−2.65	−5.23, −0.07	−0.63	0.044	−1.37	−5.19, 2.45	−0.22	0.481
% Glucose CV	41.19	42.48	1.29	−0.88, 3.46	0.36	0.243	39.98	37.67	−2.31	−5.07, 0.45	−0.51	0.101	−3.61	−7.12, −0.09	−0.63	0.044
% Glucose CV nighttime	41.13	42.37	1.24	−2.15, 4.63	0.22	0.474	40.39	35.58	−4.81	−9.28, −0.34	−0.66	0.035	−6.05	−11.69, −0.41	−0.66	0.036

Time in range, mean glucose, and glucose coefficient of variation values are presented as pertaining to the entire day or limited to nighttime hours only. Glucose concentrations are in mg/dl (to convert to mmol/L, divide by 18).

*HbA1c%* glycated hemoglobin, *TIR* time in range (70–180 mg/dl), *CV* coefficient of variation, *CI* confidence interval.

*Effect sizes (in Cohen *d*) were approximately calculated as two times *t* value divided by the square root of (sample size − 1), where *t* values were calculated as point estimates of the group difference from mixed-effects modeling divided by their robust maximum likelihood standard errors.

Though improvement in glucose sensor measures was most apparent when considered on a group-wise basis, some participants in the closed-loop group showed minimal change, likely related to participants not using the device appropriately or consistently. One participant in the CL group who showed minimal improvement in sensor glucose values also had an episode of diabetic ketoacidosis during the study; this was related to non-compliance with the use of the device. There were no other episodes of ketoacidosis during the study. In contrast, some participants in the SC group demonstrated improvement in glucose sensor indices without using a closed-loop device. Given our intent-to-treat analysis, all participants’ data were included in their study group regardless of compliance.

### Structural imaging—primary outcomes

**Fig. 1:**
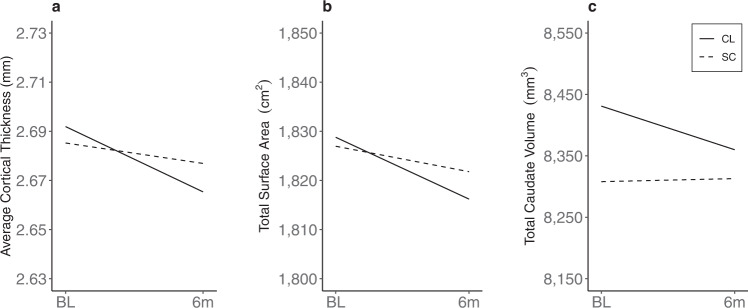
Group differences in brain structure over time. Trajectories for (**a**) average cortical thickness (mm), (**b**) total surface area (cm2), and (**c**) caudate volume (mm3) are shown for Closed Loop (CL) and Standard Care (SC) groups.

Structural neuroanatomical trajectories are readily observable in typically developing adolescents as reduced cortical gray matter volume (total and regional), cortical surface area and thickness, and increased white-matter volume^19–23^. As such, these were our primary outcome variables. In this context, between-group analyses of whole brain data derived from the *FreeSurfer®* software pipeline^24^ indicated that total cortical surface area trajectories were significantly different with the CL group showing greater reduction over time (*d* = 0.74, *P* = 0.018; Table 3 and Fig. 1). Between-group difference in average cortical thickness over time also suggested a greater reduction in the CL group (*d* = 0.58, moderate effect size). The predicted between-group difference in total gray matter reduction over time was also observed (CL > SC) though did not reach significance despite a moderate effect size (*d* = 0.42, see Table 3). Two subcortical regions were chosen for exploratory analysis; the hippocampus because of its known sensitivity to deleterious effects of dysglycemia^25,26^, and the caudate nucleus because of its extensive connectivity with the frontal lobe^27,28^, a cortical area where between-group anatomical differences were consistently observed (see below). Only total caudate volume showed significant between-group differences over time (*d* = 0.75, *P* = 0.018); the CL group showed a reduction in caudate volume over time, a trend typically observed in non-diabetic adolescents^20,21^, whereas the SC group did not.

**Fig. 2:**
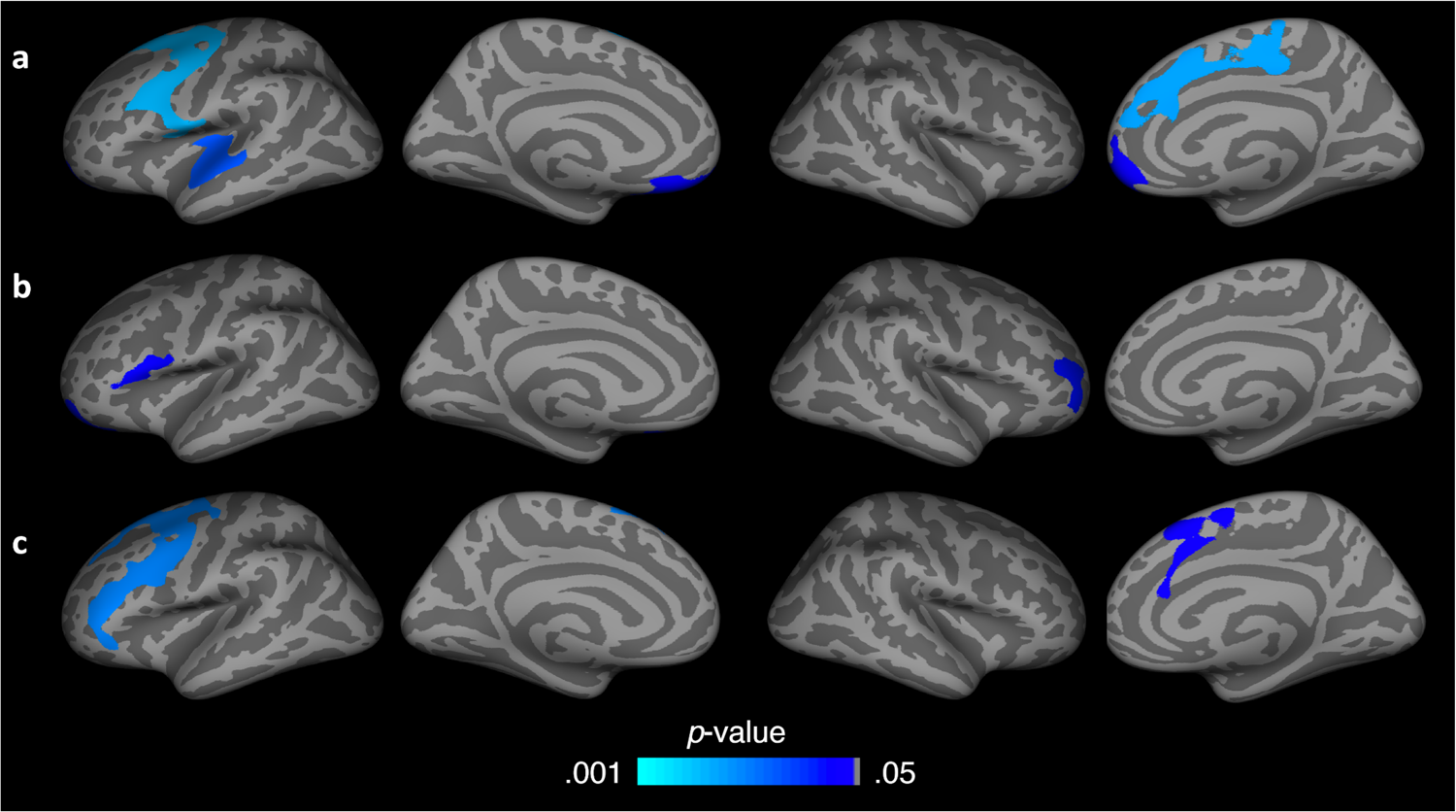
Longitudinal differences in cortical gray matter between groups. Corrected significance map showing cortical areas that exhibited a significant interaction of group by time in vertex-wise repeated measures ANOVAs that controlled for age and total brain volume in analyses of volume (**a**) and surface area (**b**), and that controlled for age in analyses of the thickness (**c**). Significance maps were thresholded using a two-tailed alpha level of 0.05, corrected for multiple comparisons. Cool colors indicate greater reductions over time in the Closed Loop (CL) group relative to the Standard Care (SC) group. The two left columns show the lateral and medial surfaces of the left hemisphere, respectively. The two right columns show the lateral and medial surfaces of the right hemisphere, respectively.

Follow-up vertex-based analyses in *FreeSurfer* were used to ascertain the regions that were most associated with overall differences in cortical morphology change over time. Results indicated that the CL group had significantly greater localized reductions in volume, surface area and thickness over time relative to the SC group within subregions of the frontal lobe (Fig. 2). Specifically, volume differences were localized to the right dorsomedial prefrontal cortex (*d* = 1.23, 95% CI: 68.47, 209.24, *P* < 0.001), right ventromedial prefrontal cortex (*d* = 1.21, 95% CI: 53.23, 167.06, *P* = 0.003), left superior temporal and insular cortex (*d* = 1.27, 95% CI: 61.34, 180.28, *P* = 0.002), left ventromedial prefrontal cortex (*d* = 1.05, 95% CI: 62.40, 248.55, *P* = 0.002), and the left dorsolateral prefrontal cortex (*d* = 1.37, 95% CI: 159.92, 426.08, *P* < 0.001). Surface area differences were localized in the left inferior frontal gyrus (*d* = 1.32, 95% CI: 5.81, 16.28, *P* = 0.001), and right frontopolar cortex (*d* = 1.24, 95% CI: 15.43, 46.85, *P* = 0.002). Thickness differences were localized to the left dorsolateral and ventrolateral prefrontal cortex (*d* = 1.59, 95% CI: 0.05, 0.11, *P* < 0.001), and the right dorsomedial prefrontal cortex (*d* = 1.43, 95% CI: 0.05, 0.12, *P* < 0.001).

**Fig. 3:**
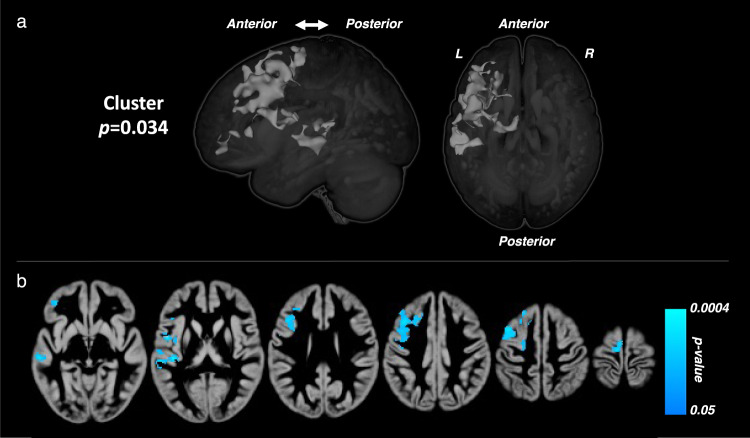
Longitudinal differences in whole-brain gray matter between groups. Brain maps resulting from voxel-based morphometry analysis showing the location of significant between-group differences in regional gray matter trajectories. Regional differences in brain volume between participants in the closed-loop (CL) and standard care (SC) groups were analyzed using voxel-wise repeated measures general linear model, covarying for average total gray matter (or white matter) volume and age. Significance maps were thresholded using a two-tailed alpha level of 0.05, corrected for multiple comparisons. **a** 3D surface rendering of the cluster (light gray) that exhibits between-group differences, corrected for multiple comparisons. **b** Voxel-wise *P* value map of gray matter growth differences within the significant cluster. Cool colors indicate greater reductions over time in the Closed Loop (CL) group relative to the Standard Care (SC) group.

Regional, between-group differences in gray matter trajectories were also interrogated with whole-brain, voxel-based morphometry using *SPM12* software (Wellcome Department of Imaging Neuroscience, University College London, London, UK, http://www.fil.ion.ucl.ac.uk/spm). After accounting for overall gray matter volume and age, these analyses showed significantly greater, age-appropriate reductions in gray matter in the CL group over time, in a cluster that encompassed the left frontal lobe and left superior temporal cortex (*d* = 0.77, 95% CI: −0.019, −0.003, *P* = 0.007; Fig. 3).

### Diffusion-weighted imaging—primary outcome

Studies of typically developing adolescents consistently report increasing white-matter fractional anisotropy over time (e.g., ref. 19). Accordingly, we used the *Tracula* module^29^ of *FreeSurfer* to determine changes in whole-brain microstructural values of fractional anisotropy (FA; primary outcome) over the 6-month duration of this study. Axial diffusivity (AD), radial diffusivity (RD), and mean diffusivity (MD) were also measured on an exploratory basis. After accounting for overall brain volume and age, the resulting analyses showed that the CL group had larger increases in average brain FA during the study period compared to the SC group (*d* = 0.68, *P* = 0.030). Given the frontal lobe focus of structural imaging findings, we examined FA changes in prominent frontal white-matter tracts, including the anterior thalamic radiation and superior longitudinal fasciculus (parietal branch). These post hoc analyses utilized automated procedures available in *Tracula* software for tract segmentation and age as covariates. Significant between-group differences in average FA over time were observed for both frontal tracts with the CL group demonstrating significantly larger increases over time (*P’s* < 0.025). Supplementary Table S1 shows changes over time in all *Tracula*-defined white-matter tracts.

### Functional magnetic resonance imaging (fMRI)—secondary outcome

**Fig. 4:**
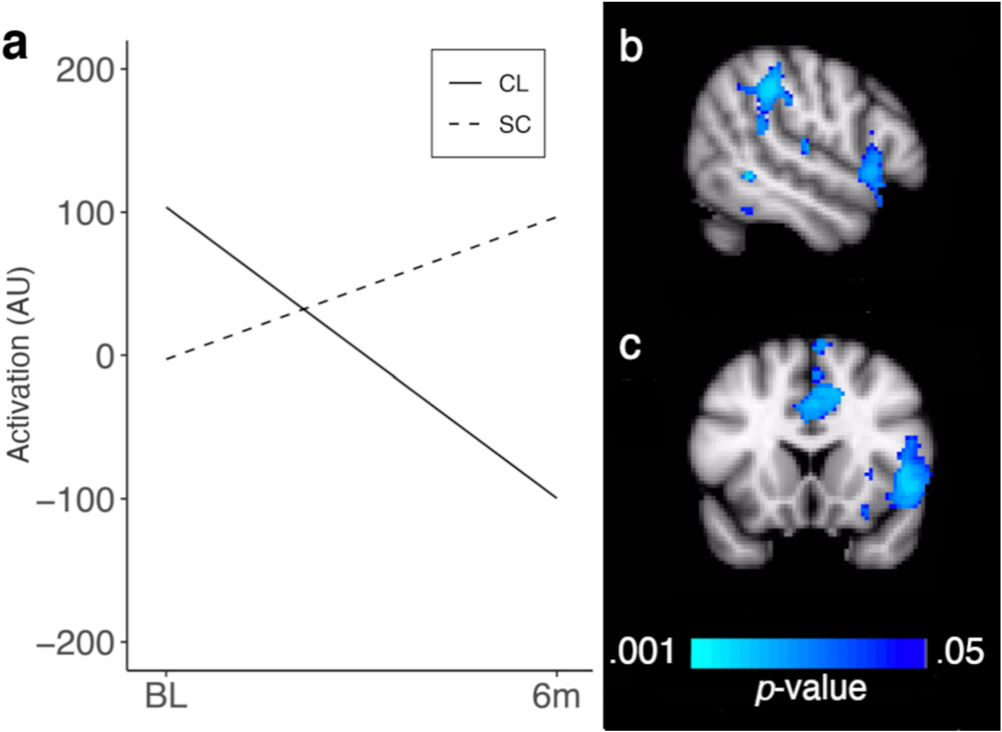
Longitudinal differences in brain activation between groups. Results from fMRI analyses showing a greater reduction in activation over time in the Closed Loop (CL) relative to the Standard Care (SC) group. **a** Line chart showing changes in regional activity over time by group based on mixed-effects modeling conditional on age (Y axis is in arbitrary units or “AU”). The right panel (**b**, **c**) shows brain areas that exhibited a significant interaction of group by time in voxel-wise linear mixed effects controlling for age. Significance maps were thresholded using a two-tailed alpha of 0.05, corrected for multiple comparisons. Cool colors indicate reduced activation over time in the CL relative to the SC group. Group by time differences were predominantly located in subregions of the executive function network, including the right inferior frontal gyrus and right parietal cortex as well as the dorsal anterior cingulate cortex. Panel **b** displays a sagittal view of the brain; panel **c** displays a coronal view.

Analyses of brain activation with *FSL* (FMRIB Software Library, version 5.0.8) during a response inhibition (Go/No-Go) task revealed a significant group by time interaction (average group difference over 6 months = 302.77 with 95% confidence interval of 193.63 to 411.91, *d* = 1.76, *P* < 0.001). This result appeared to be most influenced by a greater reduction in activation in the CL group relative to the SC group in regions subserving attention, inhibition, and executive function (e.g., the dorsal anterior cingulate, inferior frontal gyrus and parietal cortex; *P* < 0.05, TFCE FWE-corrected; Fig. 4). No significant differences in task performance were observed between the groups over time, in either accuracy of responses (as indexed by the signal detection measure, d-prime: *d* = 0.64, 95% CI = −0.15, 0.98, *P* = 0.054) or reaction time to correct Go trials (*d* = 0.08, 95% CI = −37.96, 30.37, *P* = 0.815).

### Cognitive function—secondary outcome

The CL group showed greater improvement over time in the WASI-II Perceptual Reasoning Index (PRI) score compared to the SC group (Cohen’s *d* = 0.82, *P* = 0.009). Significant between-group differences were not observed for change in the Verbal Comprehension Index (VCI) or Full-Scale IQ (FSIQ) scores (Table 2).

**Table 2:** Changes in IQ scores from baseline to 6 months and intention-to-treat (ITT) effects on these changes (all estimated based on mixed-effects modeling)

	Standard care						Closed loop						Group difference in change			
	Baseline	6 months	Change	95% CI	Effect size*	*P* value	Baseline	6 months	Change	95% CI	Effect size*	*P* value	Change	95% CI	Effect size*	*P* value
Perceptual Reasoning Index	109.29	111.33	2.05	−0.17, 4.27	0.57	0.070	108.05	114.14	6.10	4.05, 8.14	1.82	0.000	4.05	1.03, 7.07	0.82	0.009
Verbal Comprehension Index	112.38	112.67	0.29	−2.89, 3.47	0.05	0.860	110.24	111.71	1.48	−0.87, 3.82	0.39	0.217	1.19	−2.76, 5.14	0.18	0.555
Full-Scale IQ	111.81	113.76	1.95	−0.22, 4.13	0.55	0.078	110.19	114.24	4.05	1.98, 6.11	1.20	0.000	2.10	−0.90, 5.09	0.43	0.171

*CI* confidence interval.

*Effect sizes (in Cohen *d*) were approximately calculated as two times *t* value divided by the square root of (sample size − 1), where t values were calculated as point estimates of the group difference from mixed-effects modeling divided by their robust maximum likelihood standard errors.

**Fig. 5:**
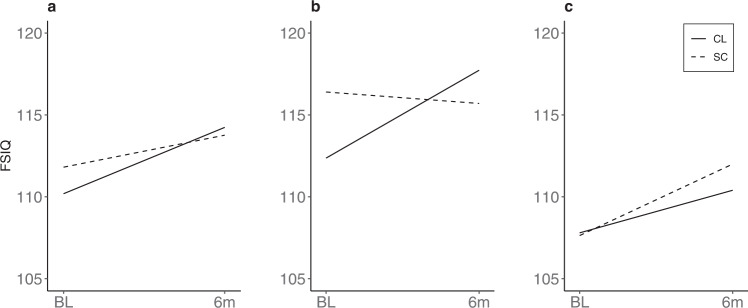
Moderator of treatment effect on cognitive trajectories. % Glucose >250 mg/dl as a moderator of treatment effect on Full-Scale IQ (FSIQ): **a** overall intention-to-treat effect in the total sample. **b** Baseline % glucose >250 mg/dl < =25%. **c** Baseline % glucose >250 mg/dl >25%. Line charts show change from baseline (BL) to end of study at 6 months (6 m). Trajectories for Closed Loop (CL) and Standard Care (SC) groups are shown.

As an exploratory/secondary analysis, demographic and baseline glucose variables were examined as potential moderators of treatment effect on cognitive trajectories. Using a median split, %baseline glucose > 250 mg/dl was observed to be a significant moderator of treatment effects on VCI (*P* = 0.008) and FSIQ (*P* = 0.005) for this interaction (Fig. 5). That is, participants in the CL group who started with lower values, indicating better glucose control (≤25% of sensor glucose >250 mg/dl), showed greater change in scores over time compared to the SC group (VCI: *d* = 0.69, *P* = 0.026; FSIQ: *d* = 0.97, *P* = 0.002), whereas those who started with higher %glucose >250 mg/dl (>25%) did not (Fig. 5b/c) (VCI: *d* = −0.47, *P* = 0.134; FSIQ: *d* = −0.27, *P* = 0.384). Similarly, median split of baseline %TIR Nighttime was also found to be a significant moderator of treatment effects on VCI (*P* = 0.029). That is, participants in the CL group with higher %TIR at nighttime (>37%) at baseline benefited more from the CL intervention, whereas those who started with lower values (≤37%) did not (VCI: *d* = −0.30, *P* = 0.331). Treatment effects for VCI and FSIQ were not detectable in the overall intent-to-treat analyses with the total sample when the two heterogeneous effects are averaged (Fig. 5a). No significant glycemic moderators were identified for PRI.

### Association of change in glucose with change in cognitive function and imaging metrics

**Fig. 6:**
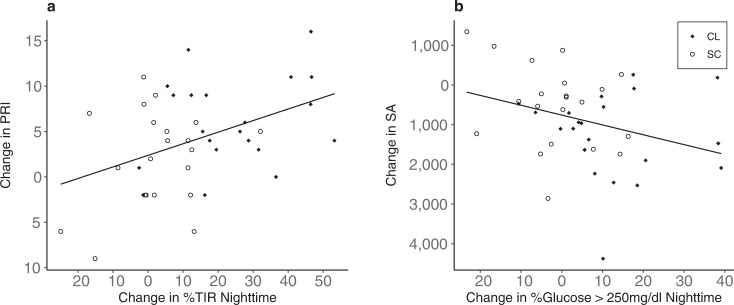
Correlation between change in glucose sensor values and change in cognitive and imaging metrics over time. Association of change from baseline to 6 months in (**a**) Perceptual Reasoning Index (PRI) with % time in range (TIR) nighttime and (**b**) cortical surface area (SA) with % glucose >250 mg/dl nighttime. Closed Loop (CL) group shown by solid diamonds, Standard Care (SC) groups by open circles.

Post hoc, exploratory analyses (without correction for multiple comparisons) were performed using the entire T1D sample (combining both the CL and SC groups) to generate hypotheses for future studies. Bivariate correlations were employed to determine if significant associations existed between changes in glucose sensor values and changes in cognitive or imaging metrics. These exploratory analyses assessed the potential association between prespecified glycemic variables of interest in this study (i.e., TIR, % glucose >250 mg/dl), with cognitive or brain variables showing significant between-group differences in the topline analyses (i.e., PRI, SA). Increase in PRI over time was significantly correlated with higher nighttime %TIR (*r* = 0.42, *P* = 0.005; Fig. 6a) as well as full day %TIR (*r* = 0.35, *P* = 0.020). Similarly, gains in PRI were negatively correlated with change in % glucose >250 mg/dl nighttime (*r* = −0.40, *P* = 0.008) and full day (*r* = −0.32, *P* = 0.040).

With respect to associations between glycemia and measures of brain structure and function, change in % glucose >250 mg/dl and change in cortical surface area (SA) were significantly correlated such that lower sensor values over time (i.e., better control) during both nighttime (*r* = 0.32, *P* = 0.039) and full day (*r* = 0.31, *P* = 0.048) were associated with reduced SA (Fig. 6b). With regard to glucose variation, reduced caudate volume over time was significantly correlated with reduced nighttime glucose CV (*r* = 0.36, *P* = 0.031). The correlation between full-day glucose CV and reduced caudate volume showed a similar effect but did not reach significance (*r* = 0.32, *P* = 0.056). Similar associations were observed between reduced SA and reduced glucose CV over time (nighttime *r* = 0.30, *P* = 0.073; full day *r* = 0.34, *P* = 0.039).

Vertex-based, whole brain *Freesurfer* analyses using the same glucose sensor variables showed significant associations between regional (primarily frontal) change in SA and both %TIR (full day) and % glucose >250 mg/dl (full day and nighttime; see Supplementary Fig. S1).

## Discussion

Research results from the DirecNet consortium and other investigators indicate that hyperglycemia has measurable, deleterious effects on MRI metrics of brain development and standardized scores on tests of cognitive and neuropsychological function in children and adolescents with T1D^5,6,18^. At the same time, it is well-established that typical puberty and adolescence is characterized by dynamic changes in brain structure and function highlighted by demonstrable remodeling of cortical and subcortical anatomy. Using multimodal brain imaging techniques, these changes are observable in typically developing adolescents as reduced cortical gray matter volume, subcortical tissue volume, cortical surface area and thickness, and increasing white-matter volume and fractional anisotropy^19–23^. Developmental alterations to frontal–striatal networks during adolescence are particularly pronounced and are thought to underlie cognitive changes during this period^30–32^.

In this preliminary, proof-of-concept study of adolescents with T1D, we found that participants who were randomized to wearing a hybrid, closed-loop device over 6 months showed significant reductions in hyperglycemia and measures of glucose variation, relative to adolescents randomized to standard diabetes care. These between-group differences were accompanied by gains in standardized IQ scores and multiple metrics of brain development and function strongly indicating a tendency towards “normalization” in the CL group relative to the SC group.

The observed increase in the Perceptual Reasoning Index (PRI) over 6 months in the CL group (~6 points) is noteworthy. The WASI-II PRI score reflects nonverbal abilities and visuomotor skills and, as such, is a measure of a person’s ability to reason and think flexibly. Cognitive test scores of this type are typically stable during childhood and adolescence^33,34^ and thus a 6-point increment (Table 2) is likely meaningful and outside the expected variation associated with measurement error^35^. The VCI score, a measure of crystallized abilities (stored knowledge and past experiences) and FSIQ did not change differently between groups when analyzed with an intention-to-treat approach. However, both significantly improved over time in a CL subgroup defined by better glycemic control at baseline relative to a SC subgroup who also demonstrated better baseline control (Fig. 1). This finding suggests that adolescents with T1D who have better long-term glycemic control may have a greater capacity to improve standardized IQ scores in response to interventions such as hybrid closed-loop devices. This finding also emphasizes the potential importance of rigorous glycemic control throughout a child’s lifetime.

Similar to higher standardized IQ scores, more robust decreases in cortical and subcortical metrics (cortical SA, frontal and caudate volumes) accompanied by increased white-matter FA in the CL group are all indicators of brain development more congruent with non-diabetic adolescent populations^19–23^. We also note that the frontal–superior temporal cortical cluster identified in the VBM analysis here is spatially overlapping with a similar cluster observed in a previous analysis of much younger children with T1D versus healthy controls^15^. In this previous study, the frontal–superior temporal cluster was observed to be significantly larger in the T1D group. Here, we show that a frontal–superior temporal cluster shows a steeper reduction in gray matter volume in the CL group in an older T1D cohort over a 6-month period, suggesting an anatomical trend towards that of non-diabetic adolescents.

The reduction in activation that we observed in the closed-loop group likely reflects a normalization of function in areas of the brain subserving attention and response inhibition. In a previous sample of 93 children with T1D and 57 controls scanned using the Go/No-Go task, we found anomalous increases in activation in executive control regions in the T1D group, despite equivalent accuracy and response times^11^. This suggests compensatory increases in activation of executive control networks may counteract T1D-associated abnormalities in the brain, to facilitate normative behavioral performance. The reductions in executive network function that we observed in the closed-loop group, therefore, are striking in that they indicate tighter glucose control is associated with a reduction in compensatory hyperactivation.

Though the glycemic effects of the hybrid closed-loop device used in this study were robust, they were not universal, and depended largely on the % use of the device in auto mode (closed-loop). Similarly, some participants in the SC group showed glycemic improvement, even without closed-loop technology, that was better than their group’s average sensor values. Thus, by merging the two groups into a single cohort we were able to boost overall group size and attain larger variation in glycemic variables for post hoc, exploratory analyses. When considered across the entire cohort, reductions in hyperglycemia were significantly correlated with higher standardized IQ scores and decreased cortical SA over the 6-month time span of this study regardless of group assignment. Improvement in glucose variability across the entire cohort was also associated with brain changes, including increased overall FA and reduced caudate volume as well as regional reductions in SA, all congruent with expected brain development in non-diabetic adolescents.

The results presented here underscore the importance of assessing frontal cortical brain development in adolescents with T1D. While overall indices of brain development such as SA and FA showed significant between-group differences, analyses assessing regional changes over time (i.e., VBM, *Tracula*, *FreeSurfer*), as well as fMRI, converged on the frontal lobe as a critical area for adverse effects of chronic dysglycemia. Supporting the importance of the frontal cortical results were findings that expected reductions in caudate nucleus volumes were more prominent in the CL group and were correlated with decreases in glucose variability across the entire cohort. The caudate nucleus is a major component of the corpus striatum, and frontal–striatal networks underlie the development of critical cognitive functions that undergo maturation during adolescence and young adulthood^30–32^. Given prior work from our group showing slower growth of the hippocampus over 18 months is associated with increased exposure to hyperglycemia and with greater glycemic variability^36^ it is unclear why significant effects were not observed in this area. One possibility is that changes in this region are gradual. Future studies that track children over a longer period of time would be helpful in testing this hypothesis.

Limitations of this study include small group size, proof-of-concept design, and lack of a non-diabetic healthy control group to which changes with improved glucose control can be indexed in adolescents with T1D. With respect to sample size, analyses focused on two of our global endpoints (total gray matter volume and cortical thickness) did not reach significance despite moderate between-group effect size for both variables (Table 3). We also allowed different CGMs to be used in the study, including Dexcom® G5 and G6 users, and those that did not have one were provided the iPro2 which used an Enlite® sensor provided in kind by Medtronic. Although the mean average relative difference in sensor readings was higher for the Enlite sensor (13.6%) vs either Dexcom G5 or G6 (9%), the findings reported here we believe are robust as each patient was tested pre and post 6 months follow-up using the same system—so there was no switching of sensors through the study. One participant in the CL group who showed minimal improvement in sensor glucose values had an episode of diabetic ketoacidosis during the study. Sensitivity analyses performed after excluding this participant resulted in identical findings with respect to significant between-group differences with the exception that an additional regional DTI FA change measure (superior longitudinal fasciculus-temporal branch) changed from non-significant (*d* = 0.61, *P* = 0.05) to significant (*d* = 0.70, *P* = 0.016) for CL > SC.

**Table 3:** Changes in Freesurfer-derived brain metrics (see methods) from baseline to 6 months and intention-to-treat (ITT) effects on these changes (all estimated based on mixed-effects modeling)

	Standard care						Closed loop						Group difference in change			
	Baseline	6 months	Change	95% CI	Effect size*	*P* value	Baseline	6 months	Change	95% CI	Effect size*	*P* value	Change	95% CI	Effect size*	*P* value
Gray matter volume (cm^3^)	739.16	734.33	−4.83	−8.92, −0.74	−0.72	0.021	727.27	717.96	−9.31	−14.46, −4.17	−1.11	0.000	−4.48	−11.02, 2.06	−0.42	0.179
White-matter volume (cm^3^)	485.52	485.93	0.41	−1.17, 1.99	0.16	0.610	481.50	481.53	0.03	−1.76, 1.82	0.01	0.971	−0.38	−2.79, 2.03	−0.10	0.760
Average cortical thickness (mm)	2.69	2.68	−0.01	−0.02, 0.00	−0.42	0.181	2.69	2.66	−0.03	−0.04, −0.01	−1.08	0.001	−0.02	−0.04, 0.00	−0.58	0.062
Total surface area (cm^2^)	1828.96	1823.77	−5.19	−9.48, −0.90	−0.74	0.018	1826.78	1814.23	−12.56	−16.99, −8.13	−1.74	0.000	−7.37	−13.47, −1.27	−0.74	0.018
Total caudate volume (mm^3^)	8314.59	8319.92	5.34	−39.72, 50.40	0.07	0.816	8424.35	8352.77	−71.58	−114.84, −28.32	−1.01	0.001	−76.91	−139.98, −13.84	−0.75	0.017
Total hippocampal volume (mm^3^)	8433.10	8370.07	−63.04	−111.63, −14.45	−0.79	0.011	8247.51	8215.81	−31.69	−91.59, 28.21	−0.32	0.300	31.34	−46.16, 108.84	0.25	0.428

*CI* confidence interval.

*Effect sizes (in Cohen *d*) were approximately calculated as two times *t* value divided by square root of (sample size − 1), where *t* values were calculated as point estimates of the group difference from mixed-effects modeling divided by their robust maximum likelihood standard errors.

Though 6 months of improved glycemic control might be regarded as relatively short, there are many examples of human brain imaging parameters changing within a 6-month period (or less) in other clinical groups receiving a targeted intervention (e.g., refs. 37–40). Further, our study results do not shed light on mechanisms underlying changes in MRI metrics of brain development and function or standardized IQ scores in our CL group. In addition to improvement in glycemic control, it is possible that effects on inflammation^41^ or microvascular integrity^42^ could also contribute to these changes.” The strengths of our study include the use of multimodal brain imaging outcomes, the length of intervention (6 months), and the integration of advanced technologies to improve diabetes control as a critical methodological factor affecting brain development and function in children.

In summary, in this pilot study, we observed that the use of a hybrid closed-loop device resulted in improved glycemic control in adolescents with long-standing T1D compared to standard care (MDI and open-loop pumps). We also observed that improvement in % glucose time in range and glucose variability in adolescents is associated with quantifiable changes in PRI and SA, regardless of whether conventional or hybrid closed-loop devices were utilized during the six-month study period. The fact that significant changes can be observed over a period of six months offers hope that insults to the developing adolescent brain might be preventable or even reversible with rigorous glucose control. Further research in this critical area is needed to determine if further improvements can be attained as closed-loop systems continue to evolve, and whether intervention with these systems at an even younger age mitigates or even reverses the deleterious effects of dysglycemia on young brains.

## Methods

### Study design and ethical approval

This was a pilot, multi-center, randomized, parallel-group study to examine whether brain and cognitive indices in adolescents with T1D could be improved with better diabetes control using a hybrid closed-loop insulin delivery system (ClinicalTrials.gov identifier: NCT03569631).

The study protocol, participant information, and consent form, available safety information, participant recruitment procedures, information about payments and compensation available to participants, and documentation evidencing the investigators’ qualifications were submitted to the institutional review board at the Jaeb Center for Health Research with reciprocity at the IRB of each of the five participating clinical centers (Nemours Jacksonville, Stanford, Iowa, Washington University St Louis and Yale). The study was performed in accordance with ethical principles according to the Declaration of Helsinki (Fortaleza, October 2013), seventh revision, 64th World Medical Association General Assembly Meeting and are consistent with the International Conference on Harmonization/good clinical practice, applicable regulatory requirements, and the sponsor or its delegate’s policy on bioethics.

### Participants

Enrollment took place from March 2018 through November 2018 at the five study sites. Forty-six adolescents ranging in age from 14 to 17 years diagnosed with T1D prior to age 8 years were recruited after obtaining informed written consent from the parents/guardians and child’s assent. Participants had to be on stable insulin therapy (either multiple daily injections (MDI) or open-loop pumps) and be willing to stay on the same regimen throughout the 6 months of the study. All participants had to be in puberty (at least Tanner stage 2 breasts in girls, genitals in boys). Exclusionary criteria included the history of prematurity (≤34 weeks gestation), birth weight below 2 kg, known neurologic or psychiatric illness, and diagnosed cognitive/developmental delay. Individuals with known attention deficit hyperactivity disorder on stable medication who had participated in our previous longitudinal research^18^ were allowed to join the study. Those with concomitant hypothyroidism were also permitted to participate if they were on stable thyroid replacement and had normal thyroid function. All participants had a full physical and pubertal exam at study entry and at 3 and 6 months subsequently. A hemoglobin A1c was measured at the study site using a DCA 2000 instrument.

### Randomization

The randomization step was performed after pre-qualification, enrollment, and collection of medical background information to ensure the participant met inclusion criteria. All study participants had their CGM cognitive and MRI data collected prior to their baseline visit. Of 46 recruited participants, 44 who met all our eligibility criteria were randomized. A randomization table was prepared using the Microsoft Excel RAND function and entered into REDCap, a secure web application for building and managing online surveys and databases^43,44^. Following successful participant recruitment, selected staff at each site opened an electronic case report form, clicked a “Randomize” button, and then read the result. After the randomize button had been clicked, no further changes were possible and the participant assigned to the arm chosen by the randomize procedure. The only exception was when a new participant was recruited to replace a post-randomized dropout. In this case, the new participant was assigned to the same arm previously occupied by the dropout at a specific site. We used straightforward randomization with the 50:50 assignment ratio without using any stratification (e.g., by gender or age) because of the limited sample size at each site (6–13 participants) and for the study overall. Two participants withdrew shortly after randomization; 42 participants completed the study.

### Closed-loop hybrid device and glucose sensing

A minimum 6-day run-in phase was conducted to collect baseline sensor data. Afterward, participants were randomized to receive either a closed-loop hybrid device with 24-h sensor-augmented therapy (CL group; Medtronics 670 G^®^ insulin pump) or standard care (SC group; either MDI or open-loop pump). Participants not previously wearing a continuous glucose monitor (CGM) wore a blinded Medtronic iPro®2 Professional CGM device at baseline, 3 and 6 months for at least 6 days; participants already using sensors continued to use their home unblinded CGM (Dexcom G5 or G6) over a 6-month period. Detailed patient instructions on device use were provided by expert team personnel at each center. Glucose and device use data were downloaded at 0, 3, and 6 months in both groups and analyzed at the Jaeb Center for Health Research.

### Cognitive assessment

Prior to cognitive testing blood glucose was required to be between 70 and <200 mg/dl and either bolus insulin or oral glucose administered if necessary to reach this target. Glucose levels measured before cognitive testing were comparable between the two groups at both time points (unpaired *t* test *P’s* > 0.15). All participants were administered the Wechsler Abbreviated Scale of Intelligence second edition (WASI-II^35^). The WASI-II is composed of four subtests: Block Design, Vocabulary, Matrix Reasoning, and Similarities. In addition to a full-scale IQ (FSIQ) score, the four-subtest version of the WASI-II used in this study produces a Verbal Comprehension Index (VCI) score resulting from the Vocabulary and Similarities subtests and a Perceptual Reasoning Index (PRI) score from the Block Design and Matrix Reasoning subtests. The FSIQ, VCI, and PRI represent standardized scores with a mean of 100 and a standard deviation of 15.

### Brain image acquisition, processing, and analyses

Unsedated brain MRIs were performed using previously described desensitization protocols^45^. Prior to study testing blood glucose was required to be between 70 and <200 mg/dl and titrated as described above. Glucose levels measured before imaging were comparable between the two groups at both time points (unpaired *t* test *P’s* > 0.50). MRIs were performed on Siemens 3 T Tim Trio, or for one site, Prisma, whole-body scanners using a standard 12-channel head coil and identical imaging protocols at each site. Multi-site reproducibility of imaging data for this study is described in previous publications^15,46^ except for the one site (Washington University) that transitioned to the Prisma platform for the current protocol. Pulse sequences selected for the Prisma scanner were designed and tested to be backwardly compatible to the Tim Trio. Sagittal T1 brain images were acquired using a magnetization-prepared rapid gradient-echo (MP-RAGE) pulse sequence: TR = 2300 ms, TE = 2.98 ms, TI = 900 ms, flip angle = 9°, slice thickness = 1 mm, FOV = 25.6 cm × 25.6 cm, 160 slices, matrix = 256 × 256, voxel size = 1 × 1 × 1 mm, duration = 4:54 min. Axial diffusion-weighted images were acquired using an echo planar imaging (EPI) pulse sequence: 30 diffusion gradient directions (29 with *b* = 1000 s/mm^2^, 1 with *b* = 0 s/mm^2^), TR = 8800 ms, TE = 99 ms, flip angle = 90°, slice thickness = 2 mm, FOV = 22 cm × 22 cm, 64 slices, matrix = 110 × 110, voxel size 2 × 2 × 2 mm, duration = 4:59 min. Axial–oblique functional images were acquired during a response inhibition (Go/No-Go task) on the axis of the anterior and posterior commissures using an EPI pulse sequence: TR = 2000 ms, TE = 27 ms, flip angle = 80°, slice thickness = 4 mm, gap = 0.4 mm, FOV = 22 cm × 22 cm, 33 slices, matrix = 74 × 74, voxel size 2.97 × 2.97 × 4.4 mm, nframes = 250.

The Go/No-Go task requires that participants respond as quickly and accurately as possible, using a button press, to a high number of “go” stimuli (e.g., when they see any letter except “X”), and to suppress a prepotent response on a smaller subset of “no-go” stimuli (e.g., when they see the letter “X”). Each letter trial was presented for 250 ms and was separated from the subsequent trial with a jittered intertrial interval that ranged from 750 ms to 8750 ms, during which participants passively viewed a fixation cross. Because the task was weighted towards go stimuli (*N* = 300 trials), a prepotent tendency to respond is created and increases the inhibitory effort necessary to successfully withhold responding to no-go stimuli (*N* = 75 trials). The task was divided into two separate runs, each lasting 8.3 minutes. Accuracy of responses and response times were recorded.

Anatomical imaging data were visually inspected for head motion artifacts and then manually aligned onto the axis of the anterior and posterior commissures^47^. Voxel-based morphometry (VBM) was performed based on established methods using Statistical Parametric Mapping software (*SPM12*) in *MATLAB*^48^. Briefly, data were corrected for magnetic field inhomogeneity and were subsequently segmented into gray matter (GM), white matter (WM), and cerebrospinal fluid volumes^49^. High-dimensional registration was then performed by generating a cohort-specific template using the Diffeomorphic Anatomical Registration Through Exponentiated Lie Algebra (DARTEL) toolbox^48^. Finally, images were warped and modulated into Montreal Neurological Institute (MNI) space, downsampled to 1.5 × 1.5 × 1.5 mm voxels, and spatially smoothed using a three-dimensional 6 mm full-width-at-half-maximum (FWHM) Gaussian smoothing kernel. Difference images representing brain growth over the 6-month study interval were calculated for use in statistical analyses. Regional differences in brain volume between participants in the closed-loop (CL) and standard care (SC) groups were analyzed using voxel-wise two-sample *t* tests based on a general linear model, covarying for average total gray matter (or white matter) volume and average age. Using a voxel-wise height threshold of *P* < 0.05 (uncorrected), we report significant regional results at *P* < 0.05, corrected for family-wise-error (FWE).

Cortical surface reconstruction and volumetric segmentation of subcortical regions was performed using the recon-all pipeline in the *FreeSurfer* image analysis suite, version 6.0 (http://surfer.nmr.mgh.harvard.edu/). Visual inspection of segmentations and of the gray-white and pial surfaces were conducted by a trained analyst who was blinded to the participant group. Longitudinal processing of surface-based cortical metrics was performed using an unbiased within-participant template space and image^50^ that was created using robust, inverse consistent registration^51^. A Gaussian smoothing kernel of 15 mm was applied. Vertex-based statistical analyses of change in cortical surface area, thickness and volume were conducted using symmetrized percent change (defined as the rate with respect to the average thickness) as the dependent variable. Group (CL, SC) was entered as a factor. Age and total brain volume averaged across time points were used as covariates of non-interest in analyses of volume and surface area, and averaged age was used as a covariate of non-interest in analyses of thickness. The interaction of group by time on the cortical surface area, thickness and volume was identified in corrected significance maps, thresholded using a two-tailed alpha level of 0.05. Correction for multiple comparisons was conducted using Monte Carlo Null-Z simulation^52^.

Diffusion-weighted imaging data quality was assessed via DTIPrep software^53^ to ensure a minimum of 27 usable diffusion gradient directions per volume. Global probabilistic tractography was then performed using TRActs Constrained by UnderLying Anatomy (TRACULA)^29^ within the *FreeSurfer* 6.0 image analysis suite. Briefly, diffusion volumes were corrected for eddy-current distortions and were aligned to the T1-weighted structural images that were previously segmented. White-matter fiber tract locations were then computed for 18 tracts by a maximum likelihood estimate using the ball-and-stick model at each voxel^54^ combined with a priori knowledge of tract locations based on prior distributions on the neighboring anatomical structures. Standard diffusion measures, including fractional anisotropy (FA), axial diffusivity (AD), radial diffusivity (RD), and mean diffusivity (MD), were calculated based on the average value of voxels with >20% of the maximum probability within the highest probability 1-D path for each tract. Summary measures for eight interhemispheric tracts were calculated as volume-weighted combinations of left and right tracts. Global measures were similarly calculated as a volume-weighted combination of all tracts, excluding forceps major and minor.

Preprocessing of functional MRI (fMRI) data was conducted in *FSL* (FMRIB Software Library), version 5.0.8, with *FEAT* (FMRI Expert Analysis Tool), using methods previously described^11^. Timepoint-specific activation summary maps for the no-go correct minus go correct (“no-go >go”) contrast were computed separately for each participant and carried to higher-level voxel-based analyses. The interaction of group by time, controlling for average age was examined using the Sandwich Estimator^55^. Corrected significance maps of the interaction of group by time on activation were computed using FSL’s randomize permutation tool^56^; this approach uses a threshold-free cluster enhancement (TFCE) procedure, and a correction for family-wise error (*P* < 0.05) with 10,000 iterations.

### Blinding

All research staff scoring the cognitive assessments as well as those processing imaging data (before statistical analysis) were blinded to group status until completion of the study. Because of differences in appearance and instructional requirements of the closed-loop device versus standard care, participants and their families were not blinded to group status.

### Outcomes

Our main hypothesis was that larger reduction of hyperglycemia in the CL group, relative to the SC group, would result in greater normalization towards typical adolescent development over time in key brain metrics assessed at baseline and end of study (6 months). These primary endpoints consisted of total cortical gray and white-matter volumes, total cortical surface area, average cortical thickness, average fractional anisotropy (FA, a measure of white-matter microstructure) and regional gray matter metrics derived from voxel-based (i.e., VBM) and vertex-based (*FreeSurfer*) analyses. The secondary hypothesis was that relative to the SC group, the CL group would show higher standardized IQ scores and functional brain activity (measured with functional MRI) more indicative of neurotypical development when measured at baseline and end of study (6 months). Finally, we conducted post hoc analyses to determine if improvements in key indices of hyperglycemia, specifically, time in range (glucose between 70 and 180 mg/dl) and percent glucose >250 mg/dl within the entire participant cohort (i.e., regardless of group assignment) would be associated with improvement in brain and cognitive metrics. Nighttime glucose sensor measurements were emphasized in these analyses as this is the period when glucose concentrations are most likely to improve while using a hybrid closed-loop system.

### Statistical methods

The primary interest of this pilot study was to estimate the intention-to-treat effect (CL vs. SC) on the change (slope) in outcomes from baseline to 6 months. We employed longitudinal mixed-effects modeling of repeatedly measured outcomes as our primary analysis strategy. All participants who completed required baseline procedures and data acquisition were randomized either to the SC or the CL condition and included in the analyses following the intention-to-treat principle. We assumed a linear trend for outcomes with two repeated measures (baseline and 6 months). We allowed a nonlinear trend using piecewise modeling for outcomes with three repeated measures (baseline, 3, and 6 months). For all outcomes, we used random intercept modeling, allowing for individual variation at baseline. We used maximum likelihood estimation implemented in Mplus version 8.4^57^. In line with the intention-to-treat principle, we utilized all available cases as long as they had at least one outcome measure under the assumption that data are missing at random conditional on observed information^58^. The results of mixed-effects modeling of key outcomes are provided in Tables 1, 2, and 3.

Initial planned sample size (*n* = 50, 25 per group) was based on an estimated effect size of *d* = 0.5 at post-treatment (6 months) as the lower bound of a clinically meaningful outcome. Under this scenario, using the proposed mixed-effects modeling and piecewise growth parametrization, the estimated power to detect treatment effect (intention to treat) is 0.83 (two-tailed, *α* = 0.05) with anticipated 15% participant attrition. Given the preliminary nature of the proposed study, we did not adjust the significance level for multiple testing in our power estimation. Due to funding and time limitations, we were able to recruit only 46 participants, 44 of whom were randomized and 42 of whom completed the study (two randomized participants failed to complete all baseline requirements and therefore were not included in the analyses).

Analysis of sensor glucose was performed using SAS software, including mean sensor glucose concentrations, CV, % time-in-range (TIR) daytime and nighttime (10 PM–6 AM), % glucose >250 mg/dl and <70 mg/dl.

For neurocognitive outcomes (PRI, VCI, FSIQ scores) and glycemic control measures (% TIR, % glucose >250 mg/dl), we conducted mixed-effects modeling without conditioning on any covariates. For total gray and white-matter volumes based on *Freesurfer*, brain activation measures based on fMRI, and DTI measures, the analyses were conducted conditional on average age (average of 3 and 6 months). For the rest of *Freesurfer* measures (average cortical thickness, total surface area, total caudate volume, total hippocampal volume), we conducted mixed-effects modeling conditional on average age and total brain volume.

We examined demographic and baseline glucose variables as potential moderators of treatment effect on gains in standardized IQ scores. For this investigation, we employed the MacArthur framework for moderator analysis^59,60^ embedded in mixed-effects modeling, following the eligibility and analytical criteria for determining moderators. For easier interpretation of moderator effects, we dichotomized continuous moderators using median splits.

Variables included in the post hoc, exploratory correlation analyses were selected a priori and restricted to those that most distinguished the CL group from the SC group. This included two glucose sensor measures as predictors (%TIR, glucose > 250%, glucose CV), PRI, and specific measures of brain structure and white-matter microanatomy with significant overall or regional between-group differences (thickness, surface area, FA, VBM cluster and caudate volumes, and fMRI beta values). Bivariate correlations used delta values derived from subtracting the baseline value from the 6-month value for measures performed at only two time points (e.g., 6-month PRI minus BL PRI). Improvement in glucose sensor values for participants in the CL principally occurred within the first 3 months of the study (see Supplementary Fig. S2). This improvement remained consistent for the remaining 3 months of the study for most CL participants. In contrast, average sensor values for most participants in the SC group changed minimally during the study period. To accommodate those few participants in either group who diverged from this pattern (i.e., variation between 3 and 6-month sensor values), delta values for sensor data were calculated as the mean of the 3- and 6-month values minus baseline in order to capture a participant-specific estimate of the “average” change from baseline. When sensor measurements were missing for either the 3- or 6-month values (one to two participants in each group depending on the measure), the remaining value was used in calculating the delta. Finally, we utilized the built-in, vertex-based, longitudinal statistical functions available in *FreeSurfer* to test if the key sensor values noted above mapped onto regional volume, thickness or surface area.

### Reporting summary

Further information on research design is available in the Nature Research Reporting Summary linked to this article.

## Supplementary information

Supplementary Information

Reporting Summary

## Data Availability

The study protocol and de-identified participant raw or processed data that support the findings of this study are available for additional analyses upon reasonable request from researchers based at academic or scientific organizations to the corresponding author (A.L.R.) or senior author (N.M.) for 2 years after the publication of this manuscript. These data are not publicly available due to them containing information that could compromise research participant privacy (e.g., MRI scans).
