# Supplementary material for: A Pilot randomized trial to examine effects of a hybrid closed-loop insulin delivery system on neurodevelopmental and cognitive outcomes in adolescents with type 1 diabetes
Source: Nat Commun. 2022 Aug 30;13:4940. doi: 10.1038/s41467-022-32289-x (PMC9427757; doi:10.1038/s41467-022-32289-x)
Supplement: Supplementary file 1 — Supplementary Information [file 41467_2022_32289_MOESM1_ESM.docx]

**Supplementary Information**


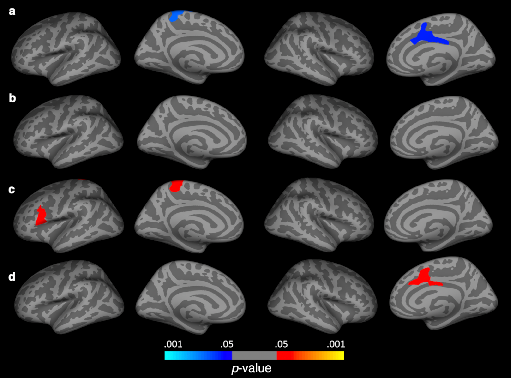


Figure S1: Correlation between change in glucose sensor values and change in cortical gray matter metrics over time. Brain maps resulting from vertex-wise repeated measures ANOVAs across the entire cohort showing regions where changes in surface area over six-months were significantly correlated with (a) %Time In Range during the full day, (b) % Time In Range nighttime, (c) %Glucose>250mg/dl for the full day and (d) %Glucose>250mg/dl nighttime. Significance maps were thresholded using a two-tailed alpha level of 0.05, corrected for multiple comparisons. Warm colors indicate significant positive associations between change in brain structure and change in glucose metric, cool colors indicate inverse associations. Analyses controlled for average total brain volume and age. The two left columns show the lateral and medial surfaces, respectively, of left hemisphere, whereas the two right columns show the lateral and medial surfaces, respectively, of the right hemisphere.

Figure S2: Change in % glucose sensor values by group at baseline (BL), 3 and 6 months. (a) % Time In Range (TIR), (b) %glucose >250mg/dl. Most change in Closed Loop (CL) group occurred during the first three months. The Standard Care (SC) group showed little change over time. Estimated trajectories based on mixed effects modeling.

| **Table S1: Changes in fractional anisotropy (FA) values of individual tracts from baseline to 6 months (tract definitions as per Tracula; see methods) and ITT effects on these changes (all estimated based on mixed effects modeling). All diffusion measures are based on the average value of voxels with > 20% of the maximum probability within the highest probability 1-D path for each tract. Intrahemispheric tracts (i.e., all except for the corpus callosum forceps major and minor) are reported as a volume-weighted combination of bilateral tracts. Analyses controlled for average age and total brain volume. FA values are presented multiplied by 100 to improve precision and interpretability.** | | | | | | | | | | | | | | | | | | | | | | | |
| --- | --- | --- | --- | --- | --- | --- | --- | --- | --- | --- | --- | --- | --- | --- | --- | --- | --- | --- | --- | --- | --- | --- | --- |
|  | *Standard Care* | | | | | | | | *Closed Loop* | | | | | | | | *Group Difference in Change* | | | | | | |
|  | Baseline | 6 months | Change | 95% CI | | | Effect Size* | p-val | Baseline | 6 months | Change | 95% CI | | | Effect Size* | p-val | Change | 95% CI | | | Effect Size* | p-val |  |
| **Anterior Thalamic Radiation** | 8.45 | 8.13 | -0.32 | -0.70 | , | 0.06 | -0.51 | 0.100 | 8.70 | 8.13 | -0.57 | -1.06 | , | -0.08 | -0.72 | 0.022 | -0.25 | -0.87 | , | 0.37 | -0.25 | 0.424 |  |
| **Superior Longitudinal Fasciculus – Parietal** | 42.72 | 44.45 | 1.72 | -3.83 | , | 7.28 | 0.19 | 0.543 | 39.60 | 57.75 | 18.15 | 12.59 | , | 23.72 | 2.00 | 0.000 | 16.43 | 8.58 | , | 24.28 | 1.28 | **0.000** |  |
| Superior Longitudinal Fasciculus – Temporal | 40.09 | 43.00 | 2.91 | -4.63 | , | 10.45 | 0.24 | 0.449 | 39.95 | 64.11 | 24.16 | 16.82 | , | 31.49 | 2.02 | 0.000 | 21.24 | 10.71 | , | 31.78 | 1.23 | **0.000** |  |
| Forceps major | 196.35 | 195.78 | -0.57 | -11.72 | , | 10.57 | -0.03 | 0.920 | 205.84 | 173.09 | -32.75 | -45.82 | , | -19.69 | -1.53 | 0.000 | -32.18 | -49.27 | , | -15.09 | -1.15 | **0.000** |  |
| Forceps minor | 25.81 | 26.37 | 0.56 | -4.51 | , | 5.62 | 0.07 | 0.830 | 29.12 | 14.26 | -14.87 | -21.33 | , | -8.41 | -1.41 | 0.000 | -15.42 | -23.60 | , | -7.24 | -1.15 | **0.000** |  |
| Cingulum - angular bundle | 24.73 | 25.49 | 0.75 | -5.31 | , | 6.82 | 0.08 | 0.808 | 23.71 | 12.20 | -11.52 | -17.83 | , | -5.20 | -1.12 | 0.000 | -12.27 | -21.02 | , | -3.52 | -0.86 | **0.006** |  |
| Cingulum - cingulate gyrus endings | 3.70 | 3.35 | -0.35 | -1.92 | , | 1.22 | -0.14 | 0.664 | 3.34 | 3.88 | 0.54 | -0.68 | , | 1.76 | 0.27 | 0.385 | 0.89 | -1.10 | , | 2.88 | 0.27 | 0.381 |  |
| Corticospinal tract | 7.02 | 5.74 | -1.28 | -4.06 | , | 1.50 | -0.28 | 0.368 | 5.50 | 2.85 | -2.65 | -5.23 | , | -0.07 | -0.63 | 0.044 | -1.37 | -5.19 | , | 2.45 | -0.22 | 0.481 |  |
| Inferior longitudinal fasciculus | 41.19 | 42.48 | 1.29 | -0.88 | , | 3.46 | 0.36 | 0.243 | 39.98 | 37.67 | -2.31 | -5.07 | , | 0.45 | -0.51 | 0.101 | -3.61 | -7.12 | , | -0.09 | -0.63 | **0.044** |  |
| Uncinate fasciculus | 41.13 | 42.37 | 1.24 | -2.15 | , | 4.63 | 0.22 | 0.474 | 40.39 | 35.58 | -4.81 | -9.28 | , | -0.34 | -0.66 | 0.035 | -6.05 | -11.69 | , | -0.41 | -0.66 | **0.036** |  |
| *Effect sizes (in Cohen d) were approximately calculated as 2 times t value divided by square root of (sample size - 1), where t values were calculated as point estimates of the group difference from mixed-effects modeling divided by their robust maximum likelihood standard errors. | | | | | | | | | | | | | | | | | | | | | | |  |

Table S1: Changes in fractional anisotropy (FA) values of individual tracts from baseline to 6 months (tract definitions as per Tracula; see methods) and Intention To Treat (ITT) effects on these changes (all estimated based on mixed effects modeling). All diffusion measures are based on the average value of voxels with > 20% of the maximum probability within the highest probability 1-D path for each tract. Intrahemispheric tracts (i.e., all except for the corpus callosum forceps major and minor) are reported as a volume-weighted combination of bilateral tracts. Analyses controlled for average age and total brain volume. FA values are presented multiplied by 100 to improve precision and interpretability. **Footnote:** *Effect sizes (in Cohen d) were approximately calculated as 2 times t value divided by square root of (sample size - 1), where t values were calculated as point estimates of the group difference from mixed-effects modeling divided by their robust maximum likelihood standard errors. CI=confidence interval.
