# Supplementary material for: A Pilot randomized trial to examine effects of a hybrid closed-loop insulin delivery system on neurodevelopmental and cognitive outcomes in adolescents with type 1 diabetes
Source: Nat Commun. 2022 Aug 30;13:4940. doi: 10.1038/s41467-022-32289-x (PMC9427757; doi:10.1038/s41467-022-32289-x)
Supplement: Supplementary file 2 — Reporting Summary [file 41467_2022_32289_MOESM2_ESM.pdf]

nature portfolio

Corresponding author(s): Allan L. Reiss, M.D.

Last updated by author(s): 3/29/2022

Reporting Summary

Nature Portfolio wishes to improve the reproducibility of the work that we publish. This form provides structure for consistency and transparency in reporting. For further information on Nature Portfolio policies, see our Editorial Policies and the Editorial Policy Checklist.

Statistics

For all statistical analyses, confirm that the following items are present in the figure legend, table legend, main text, or Methods section.

n/a	Confirmed	
☐	☒	The exact sample size (*n*) for each experimental group/condition, given as a discrete number and unit of measurement
☐	☒	A statement on whether measurements were taken from distinct samples or whether the same sample was measured repeatedly
☐	☒	The statistical test(s) used AND whether they are one- or two-sided<br>*Only common tests should be described solely by name; describe more complex techniques in the Methods section.*
☐	☒	A description of all covariates tested
☐	☒	A description of any assumptions or corrections, such as tests of normality and adjustment for multiple comparisons
☐	☒	A full description of the statistical parameters including central tendency (e.g. means) or other basic estimates (e.g. regression coefficient) AND variation (e.g. standard deviation) or associated estimates of uncertainty (e.g. confidence intervals)
☐	☒	For null hypothesis testing, the test statistic (e.g. *F*, *t*, *r*) with confidence intervals, effect sizes, degrees of freedom and *P* value noted<br>*Give P values as exact values whenever suitable.*
☒	☐	For Bayesian analysis, information on the choice of priors and Markov chain Monte Carlo settings
☒	☐	For hierarchical and complex designs, identification of the appropriate level for tests and full reporting of outcomes
☐	☒	Estimates of effect sizes (e.g. Cohen's *d*, Pearson's *r*), indicating how they were calculated

Our web collection on statistics for biologists contains articles on many of the points above.

Software and code

Policy information about availability of computer code

Data collection	Research Electronic Data Capture (REDCap)
Data analysis	SPM v12, Matlab v9, FSL v5.0.8, Freesurfer v6, DTIPrep v1.2.8

For manuscripts utilizing custom algorithms or software that are central to the research but not yet described in published literature, software must be made available to editors and reviewers. We strongly encourage code deposition in a community repository (e.g. GitHub). See the Nature Portfolio guidelines for submitting code & software for further information.

Data

Policy information about availability of data

All manuscripts must include a data availability statement. This statement should provide the following information, where applicable:

- Accession codes, unique identifiers, or web links for publicly available datasets
- A description of any restrictions on data availability
- For clinical datasets or third party data, please ensure that the statement adheres to our policy

Raw or processed data that support the findings of this study are available upon reasonable request from the corresponding author [alr] or senior author (nm). The data are not publicly available due to them containing information that could compromise research participant privacy (e.g., MRI scans).

Field-specific reporting

Please select the one below that is the best fit for your research. If you are not sure, read the appropriate sections before making your selection.

☒ Life sciences ☐ Behavioural & social sciences ☐ Ecological, evolutionary & environmental sciences

For a reference copy of the document with all sections, see nature.com/documents/nr-reporting-summary-flat.pdf

Life sciences study design

All studies must disclose on these points even when the disclosure is negative.

Sample size

Initial planned sample size (n=50, 25 per group) was based on an estimated effect size of d=0.5 at post-treatment (6 months) as the lower bound of clinically meaningful outcome. Under this scenario, using the proposed mixed effects modeling and piecewise growth parametrization, the estimated power to detect treatment effect (intention to treat) is 0.83 (two-tailed, α=.05) with anticipated 15% participant attrition. Given the preliminary nature of the proposed study, we did not adjust the significance level for multiple testing in our power estimation. Due to funding and time limitations, we were able to recruit only 46 participants, 44 of whom were randomized and 42 of whom completed the study. (Two randomized participants failed to complete all baseline requirements and therefore were not included in the analyses.)

Data exclusions

No data were excluded from the study.

Replication

This was a pilot, proof-of-concept clinical trial so no replication was attempted. Results were intended to inform future larger studies. However, the results directly build on data from DirecNet's longitudinal studies of children and adolescents with type 1 diabetes with respect to showing trends towards normalization of brain and cognitive indices in adolescents who achieved reduced hyperglycemia.

Randomization

The randomization step was performed after pre-qualification, enrollment, and collection of medical background information to ensure the participant met inclusion criteria. Of 46 recruited participants, 44 who met all our eligibility criteria were randomized. A randomization table was prepared using the Microsoft Excel RAND function and entered into REDCap, a secure web application for building and managing online surveys and databases2,3. Following successful participant recruitment, selected staff at each site opened an electronic case report form, clicked a “Randomize” button, and then read the result. After the randomize button had been clicked, no further changes were possible and the participant assigned to the arm chosen by the randomize procedure. The only exception was when a new participant was recruited to replace a post-randomized dropout. In this case, the new participant was assigned to the same arm previously occupied by the dropout at a specific site. We used straightforward randomization with the 50:50 assignment ratio without using any stratification (e.g., by gender or age) because of the limited sample size at each site (6-13 participants) and for the study overall.

Blinding

All research staff administering and scoring cognitive assessments as well as those processing imaging data (before statistical analysis) were blinded to group status until completion of the study. Because of differences in appearance and instructional requirements of the closed-loop device versus standard care, participants and their families were not blinded to group status.

Reporting for specific materials, systems and methods

We require information from authors about some types of materials, experimental systems and methods used in many studies. Here, indicate whether each material, system or method listed is relevant to your study. If you are not sure if a list item applies to your research, read the appropriate section before selecting a response.

Materials & experimental systems

n/a	Involved in the study	
☒	☐	Antibodies
☒	☐	Eukaryotic cell lines
☒	☐	Palaeontology and archaeology
☒	☐	Animals and other organisms
☐	☒	Human research participants
☐	☒	Clinical data
☒	☐	Dual use research of concern

Methods

n/a	Involved in the study	
☒	☐	ChIP-seq
☒	☐	Flow cytometry
☐	☒	MRI-based neuroimaging

Human research participants

Policy information about studies involving human research participants

Population characteristics

Forty-six adolescents ranging in age from 14 to 17 years diagnosed with T1D prior to age 8 years were recruited after obtaining informed written consent from the parents/guardians and child’s assent. Participants had to be on stable insulin therapy (either multiple daily injections (MDI) or open-loop pumps) and be willing to stay on the same regimen throughout the 6 months of the study. All participants had to be in puberty (at least Tanner stage 2 breasts in girls, genitals in boys). Exclusionary criteria included history of prematurity (≤34 weeks gestation), birth weight ≥2 kg, known neurologic or psychiatric illness, and diagnosed cognitive/developmental delay. Individuals with known attention deficit hyperactivity

	disorder on stable medication who had participated in our previous longitudinal research were allowed to join the study. Those with concomitant hypothyroidism were also permitted to participate if they were on stable thyroid replacement and had normal thyroid function. All subjects had a full physical and pubertal exam at study entry and at 3 and 6 months subsequently. A hemoglobin A1c was measured at the study site using a DCA 2000 instrument. Participant enrollment and follow-up took place from March 2018 through June 2020 at the five study sites (Stanford, Nemours, Yale, Iowa and Washington University). A total of 46 participants were enrolled. Two enrolled participants did not meet inclusion/exclusion criteria and were screen failures before randomization. Two additional participants failed to complete all required baseline assessments or procedures. The remaining 42 subjects participated in the study and were included in all data analyses in line with the intention to treat principle. There were no additional participant losses. The study was ended once all 42 subjects completed their 6-month assessment.
Recruitment	Participants were recruited from pediatric diabetes clinics at each of the participating sites (Nemours Jacksonville, Stanford, Iowa, Washington University St Louis and Yale). Thus, this sample may be biased in terms of not fully representing a community sample of adolescents with type 1 diabetes. Participant enrollment and follow-up took place from March 2018 through June 2020. A total of 46 participants were enrolled. Two enrolled participants did not meet inclusion/exclusion criteria and were screen failures before randomization. Two additional participants failed to complete all required baseline assessments or procedures. The remaining 42 subjects participated in the study and were included in all data analyses in line with the intention to treat principle. There were no additional participant losses. The study was ended once all 42 subjects completed their 6-month assessment.
Ethics oversight	The study protocol, participant information and consent form, available safety information, participant recruitment procedures, information about payments and compensation available to participants and documentation evidencing the investigators' qualifications were submitted to the institutional review board at the Jaeb Center for Health Research with reciprocity at the IRB of each of the five participating clinical centers (Nemours Jacksonville, Stanford, Iowa, Washington University St Louis and Yale).

Note that full information on the approval of the study protocol must also be provided in the manuscript.

Clinical data

Policy information about clinical studies

All manuscripts should comply with the ICMJE guidelines for publication of clinical research and a completed CONSORT checklist must be included with all submissions.

Clinical trial registration	ClinicalTrials.gov Identifier NCT0342893
Study protocol	Upon request from the senior author (nm)
Data collection	Participant enrollment, data collection and follow-up took place from March 2018 through June 2020 at each of the five participating clinical centers (Nemours Jacksonville, Stanford, Iowa, Washington University St Louis and Yale).
Outcomes	Our primary hypothesis was that greater reduction of hyperglycemia in the CL group, relative to the SC group, would result in greater improvement in key brain metrics (total/regional gray matter, cortical surface area and thickness, fractional anisotropy) indicative of neurotypical development during adolescence. The secondary hypothesis was that the CL group would show higher cognitive (IQ) outcomes and functional brain activity more indicative of neurotypical development relative to the SC group. Finally, we conducted post hoc analyses to determine if improvements in key indices of hyperglycemia, specifically, time in range (glucose between 70 and 180 mg/dl) and percent glucose >250 mg/dl within the entire participant cohort (i.e., regardless of group assignment) would be associated with improvement in brain and cognitive metrics. Nighttime glucose sensor measurements were emphasized in these analyses as this is the period when glucose concentrations are most likely to improve while using a hybrid closed-loop system.

Magnetic resonance imaging

Experimental design

Design type	Functional MRI used an event-related "Go-Nogo" task
Design specifications	Each letter trial was presented for 250 ms and was separated from the subsequent trial with a jittered intertrial interval that ranged from 750 ms to 8,750 ms, during which participants passively viewed a fixation cross. The task was weighted towards go stimuli (N = 300 trials) to build up a prepotent tendency to respond, thereby increasing the inhibitory effort necessary to successfully withhold responding to no-go stimuli (N = 75 trials). The task was divided into 2 separate runs, each lasting 8.3 min.
Behavioral performance measures	Accuracy of responses and response times were recorded with a MRI-compatible button box. Group differences in task performance—including reaction time for correct go trials, and the signal detection measure, d-prime—were assessed using standard analysis of variance.

Acquisition

Imaging type(s)	structural, diffusion, functional MRI
Field strength	3T
Sequence & imaging parameters	Sagittal T1 brain images were acquired using a magnetization-prepared rapid gradient-echo (MP-RAGE) pulse sequence: TR = 2,300ms, TE = 2.98ms, TI = 900ms, flip angle = 9, slice thickness = 1mm, FOV = 25.6cm x 25.6cm, 160 slices, matrix

= 256 x 256, voxel size = 1 x 1 x 1mm, duration=4:54 min. Axial diffusion-weighted images were acquired using an echo planar imaging (EPI) pulse sequence: 30 diffusion gradient directions (29 with b=1,000 s/mm2, 1 with b=0 s/mm2), TR = 8,800ms, TE = 99ms, flip angle = 90, slice thickness = 2mm, FOV = 22cm × 22cm, 64 slices, matrix = 110 × 110, voxel size 2 × 2 × 2 mm, duration=4:59 min. Axial-oblique functional images were acquired on the axis of the anterior and posterior commissures using an EPI pulse sequence: TR = 2,000ms, TE = 27ms, flip angle = 80 slice thickness = 4mm, gap = 0.4mm, FOV = 22cm × 22cm, 33 slices, matrix = 74 × 74, voxel size 2.97 × 2.97 × 4.4 mm, nframes=250, duration=8:20.

Area of acquisition: Whole brain acquisition

Diffusion MRI: ☒ Used ☐ Not used

Parameters: For DTI, 30 diffusion gradient directions (29 with b=1,000 s/mm2, 1 with b=0 s/mm2); single shell without cardiac gating.

Preprocessing

Preprocessing software

Anatomical imaging data were visually inspected for head motion artifacts and then manually aligned onto the axis of the anterior and posterior commissures. Voxel-based morphometry (VBM) was performed based on established methods using Statistical Parametric Mapping software (SPM12) in MATLAB9. Briefly, data were corrected for magnetic field inhomogeneity and were subsequently segmented into gray matter (GM), white matter (WM), and cerebrospinal fluid volumes. High-dimensional registration was then performed by generating a cohort-specific template using the Diffeomorphic Anatomical Registration Through Exponentiated Lie Algebra (DARTEL) toolbox. Finally, images were warped and modulated into Montreal Neurological Institute (MNI) space, down-sampled to 1.5 x 1.5 x 1.5 mm voxels, and spatially smoothed using a 3-dimensional 6mm full-width-at-half-maximum (FWHM) Gaussian smoothing kernel. Difference images representing brain growth over the 6-month study interval were calculated for use in statistical analyses. Regional differences in brain volume between participants in the closed loop (CL) and standard care (SC) groups were analyzed using voxel-wise two-sample t-tests based on a general linear model, covarying for average total gray matter (or white matter) volume and average age. Using a voxel-wise height threshold of $p < 0.05$ (uncorrected), we report significant regional results at $p < 0.05$, corrected for family-wise-error (FWE).

Cortical surface reconstruction and volumetric segmentation of subcortical regions was performed using the recon-all pipeline in the FreeSurfer image analysis suite, version 6.0. Visual inspection of segmentations and of the gray-white and pial surfaces were conducted by a trained analyst who was blinded to participant group. Longitudinal processing of surface-based cortical metrics was performed using an unbiased within-subject template space and image that was created using robust, inverse consistent registration. A Gaussian smoothing kernel of 15 mm was applied. Vertex-based statistical analyses of change in cortical surface area, thickness and volume were conducted using symmetrized percent change (defined as the rate with respect to the average thickness) as the dependent variable.

Diffusion-weighted imaging data quality was assessed via DTIPrep software to ensure a minimum of 27 usable diffusion gradient directions per volume. Global probabilistic tractography was then performed using TRActs Constrained by UnderLying Anatomy (TRACULA) within the FreeSurfer 6.0 image analysis suite. Briefly, diffusion volumes were corrected for eddy-current distortions and were aligned to the T1-weighted structural images that were previously segmented. White-matter fiber tract locations were then computed for 18 tracts by a maximum likelihood estimate using the ball-and-stick model at each voxel16 combined with a priori knowledge of tract locations based on prior distributions on the neighboring anatomical structures. Standard diffusion measures, including fractional anisotropy (FA), were calculated based on the average value of voxels with > 20% of the maximum probability within the highest probability 1-D path for each tract. Summary measures for eight interhemispheric tracts were calculated as a volume-weighted combinations of left and right tracts. Global measures were similarly calculated as a volume-weighted combination of all tracts, excluding forceps major and minor.

Preprocessing of functional MRI (fMRI) data was conducted in FSL (FMRIB Software Library), version 5.0.8, with FEAT (FMRI Expert Analysis Tool), using methods previously described. Briefly, the first 3 volumes of each scan were discarded to allow for stabilization of longitudinal magnetization. Non-brain material was removed from both the anatomical and functional images using the Brain Extraction Tool. Preprocessing included motion correction to the mean image, spatial smoothing using a Gaussian smoothing kernel of 6-mm FWHM, and high-pass temporal filtering.

Normalization

Spatial normalization of fMRI data was conducted using a linear registration that was performed using FMRIB's Linear Image Registration Tool to linearly align each individual's functional data to his/her high-resolution anatomical image. Nonlinear registration was used to align each individual's anatomical image to standardized space using a publicly available template in Montreal Neurological Institute (MNI) space. The linear and nonlinear transformations were combined to register each individual's functional data to template space.

For vertex-based analyses of cortical gray matter volume, thickness and surface area, as well as regional subcortical volumes, intra-subject spatial normalization was conducted using the longitudinal processing stream in FreeSurfer. This creates an unbiased within-subject template space and image using robust, inverse consistent registration. Single subject data was then spatially normalized to the FreeSurfer average subject ("FSAverage") brain.

For voxel-based morphometry, high-dimensional registration was performed by generating a cohort-specific template using the Diffeomorphic Anatomical Registration Through Exponentiated Lie Algebra (DARTEL) toolbox in SPM12. Normalization to MNI space was then performed using the MNI152 template.

Normalization template

fMRI data were spatially normalized to the MNI-152 template (https://fsl.fmrib.ox.ac.uk/fsl/fslwiki/Atlases).

Structural MRI data processed with FreeSurfer were spatially normalized to the FreeSurfer average subject ("FSAverage") brain. This template is an average of 40 subjects using a spherical averaging described in Fischl et al. (1999).

Noise and artifact removal

In fMRI analyses, motion correction parameters and time points that exceeded a motion threshold (75th percentile plus 1.5 times the interquartile range, defined by FSL's motion outliers tool; http://fsl.fmrib.ox.ac.uk/fsl/fslwiki/FSLMotionOutliers) were included as covariates of non-interest in statistical time series analyses at the single subject level.

Volume censoring

See above; fMRI time points that were corrupted by extreme motion were censored at the intra-subject level of statistical analysis using a covariate of non-interest.

Statistical modeling & inference

Model type and settings

In analyses of fMRI data, because 2 separate task runs were conducted for each participant, time-series statistical analyses were carried out at a single-run intraindividual level using a generalized linear model that modeled each condition and accuracy type (go correct, go incorrect, no-go correct, no-go incorrect) using a synthetic hemodynamic response function and its first derivative, as well as motion correction parameters and time points that exceeded a motion threshold (75th percentile plus 1.5 times the interquartile range) defined by FSL's motion outliers tool (http://fsl.fmrib.ox.ac.uk/fsl/fslwiki/FSLMotionOutliers). Both runs were combined in a fixed effects analysis for each participant to provide individual-specific summaries of activation. These time point-specific activation summary maps for the no-go correct minus go correct ("no-go > go") contrast were computed separately for each subject and carried to higher-level voxel-based analyses. The interaction of group by time, controlling for average age was examined using the Sandwich Estimator.

Vertex-based statistical analyses of change in cortical surface area, thickness and volume were conducted using symmetrized percent change (defined as the rate with respect to the average thickness) as the dependent variable. Group (CL, SC) was entered as a factor. Analyses of brain structure used average age and average total brain volume (averaged across time points) as covariates of non-interest, with exception of cortical gray matter thickness, which used average age as the only covariate of non-interest.

For voxel-based morphometry, regional differences in brain volume between participants in the closed loop (CL) and standard care (SC) groups were analyzed using mass univariate, voxel-wise, two-sample t-tests based on a general linear model, covarying for average total gray matter (or white matter) volume and average age.

We employed longitudinal mixed effects modeling of repeatedly measured outcomes as our primary analysis strategy for diffusion-weighted microstructural data generated from Tracula. Our approach assumed a linear trend for outcomes and used random intercept modeling to allow for individual variation at baseline. Age and total brain volume were used as covariates of non-interest. We used maximum likelihood estimation implemented in Mplus version 8.4 20. In line with the intention to treat principle, we utilized all available cases as long as they had at least one outcome measure under the assumption that data are missing at random conditional on observed information.

Effect(s) tested

The primary model for all neuroimaging analyses involved the interaction of group by time, examined using a two-tailed alpha level of 0.05, corrected for multiple comparisons.

Specify type of analysis: ☒ Whole brain ☐ ROI-based ☐ Both

Statistic type for inference
(See Eklund et al. 2016)

In vertex-based analyses of cortical gray matter volume, thickness and surface area, cluster-wise probability values, computed using Monte Carlo simulations were used to compute statistical significance of group by time interactions.

In voxel-based FMRI analyses, threshold free cluster enhancement (TFCE) was used to compute statistical significance of group by time interactions.

For voxel-based morphometry, regional differences in brain volume were analyzed using a voxel-wise height threshold of $p < 0.05$ (uncorrected), we report significant regional results at $p < 0.05$, corrected for family-wise-error (FWE).

Correction

In fMRI analyses, corrected significance maps of the interaction of group by time on activation was computed using FSL's randomize permutation tool; this approach uses a threshold-free cluster enhancement (TFCE) procedure, and a correction for family-wise error ($p < 0.05$) with 10,000 iterations.

Correction for multiple comparisons across surface vertices in FreeSurfer analyses of cortical gray matter volume, thickness and surface area, was conducted using a two-tailed threshold of $p<0.05$ in conjunction with the Monte-Carlo simulation toolbox. This approach estimates the probability of forming a maximum cluster of that size or larger during the simulation under the null hypothesis that results in a cluster-wise probability (CWP). Monte-Carlo simulations replace FDR (false discovery rate) and FWER (family-wise error rate) procedures that are commonly used in structural or functional paradigms to correct for multiple comparisons.

For voxel-based morphometry, we report cluster-level results, corrected for family-wise error (FWE).

No correction for the primary diffusion-weighted data analysis (i.e., fractional anisotropy, FA) was utilized since between-group differences in FA development over time was a primary, a priori hypothesis.

Models & analysis

n/a	Involved in the study
☒	☐ Functional and/or effective connectivity
☒	☐ Graph analysis
☒	☐ Multivariate modeling or predictive analysis
